# From Data to Insights: Modeling Urban Land Surface Temperature Using Geospatial Analysis and Interpretable Machine Learning

**DOI:** 10.3390/s25041169

**Published:** 2025-02-14

**Authors:** Nhat-Duc Hoang, Van-Duc Tran, Thanh-Canh Huynh

**Affiliations:** 1 Institute of Research and Development, Duy Tan University, Da Nang 550000, Vietnam; hoangnhatduc@duytan.edu.vn; 2 Faculty of Civil Engineering, Duy Tan University, Da Nang 550000, Vietnam; tranvanduc1@dtu.edu.vn; 3 International School, Duy Tan University, Da Nang 550000, Vietnam

**Keywords:** built environment, land surface temperature, urban heat, interpretable machine learning, Shapley additive explanations

## Abstract

This study introduces an innovative machine learning method to model the spatial variation of land surface temperature (LST) with a focus on the urban center of Da Nang, Vietnam. Light Gradient Boosting Machine (LightGBM), support vector machine, random forest, and Deep Neural Network are employed to establish functional relationships between urban LST and its influencing factors. The machine learning approaches are trained and validated using remote sensing data from 2014, 2019, and 2024. Various explanatory variables representing topographical and spatial characteristics, as well as urban landscapes, are used. Experimental results show that LightGBM outperforms other benchmark methods. In addition, Shapley Additive Explanations are utilized to clarify the impact of the factors affecting LST. The analysis outcomes indicate that while the importance of these variables changes over time, urban density and greenspace density consistently emerge as the most influential factors. LightGBM attained R^2^ values of 0.85, 0.92, and 0.91 for the years 2014, 2019, and 2024, respectively. The findings of this work can be helpful for deeper understanding of urban heat stress dynamics and facilitate urban planning.

## 1. Research Background and Motivations

The global urban population is expected to increase by 2.5 billion people from 2018 to 2050, with nearly 90% of this growth occurring in Asia and Africa [1]. The rapid pace of urbanization typically signifies a shift from natural landscapes to built environments; this fact leads to alterations in local climates that change both social and ecological sustainability [2]. The rapid expansion of urban areas often results in the reduction, isolation, and fragmentation of greenspaces, adversely affecting the ability of cities to provide cooling effects and other environmental benefits [3]. Consequently, rapid urban growths have led to significant changes in land cover and an inevitable increase in land surface temperature (LST) [4].

The increase in the LST urban environment compared to their rural surroundings is widely known as the Surface Urban Heat Island (SUHI) effect [5]. SUHI has emerged as a significant contributor to local, regional, and global climate change [6]. This phenomenon can lead to various negative impacts, including the increased energy demands due to greater reliance on air conditioning, the intensification of heat waves, alterations in local wind and rainfall patterns, and a rise in smog and atmospheric aerosols. As global temperatures continue to rise and urbanization accelerates, the consequences of the SUHI effect are anticipated to escalate. Consequently, SUHI has attracted substantial research interest and has been actively studied, particularly over the past decade [7,8,9,10].

In remote sensing, the advent of thermal sensors on satellite platforms has created new opportunities for assessing SUHI effects at meso- and low-resolution spatial scales. Notably, LST can be measured using remote sensing techniques and thermal infrared (TIR) sensors [11]. Thermal sensors mounted on Landsat satellites have been used to obtain LST data on a global scale [12]. In recent years, the Google Earth Engine (GEE) cloud computing platform can be used to retrieve LST data from Landsat series [13,14]. Voogt, Oke [5] pointed out that LST can be employed as a surrogate for assessing surface thermal features. Additionally, medium-resolution sensors, such as Landsat and MODIS, have proven to be essential for analyzing urban thermal conditions, particularly in regions with limited data availability [15]. The availability of publicly accessible remote sensing data for LST has significantly enhanced the ability of scholars to study urban thermal environments across various spatial and temporal scales [16].

Land cover and land use, along with their changes, are undeniably among the most significant factors influencing SUHI [7]. Critical factors, such as built-up and greenspace areas, play a crucial role in explaining the spatial and temporal characteristics of LST in urban environments [16]. The transformation of land cover due to urbanization can significantly impact urban heat stress and overall heat comfort [17]. As natural landscapes are replaced with impervious surfaces like asphalt and concrete, the ability of the environment to absorb and dissipate heat diminishes. This leads to higher surface temperatures and intensifies the SUHI, resulting in increased heat stress for residents. These facts highlight the importance of LST estimation to enhance planning and mitigation strategies.

LST predictions involve forecasting spatial variation of this variable based on historical LST data, along with other explanatory factors such as changes in land use, topographic features, density of impervious surface, greenspace coverage, proximity to water bodies, and climate patterns [3,18,19]. Capable models for LST estimations are vital for urban planning, hot spot identification, and urban heat stress mitigation. In the current literature, various predictive approaches were employed for LST modeling, including statistical models and machine learning algorithms. These methods have been shown to be capable of simulating the interactions between the variable of interest and its influencing factors [20]. Notably, spatial modeling of LST plays a crucial role in formulating strategies to identify and optimize the land cover types that have the most significant impact on cooling effects. By focusing on these key areas, urban planners and local authorities can enhance the effectiveness of their interventions.

In recent years, there has been an increasing trend of applying machine learning and Geographic Information Systems (GIS) technology in spatial estimation of LST in urban environments [21,22]. The integration of machine learning and GIS offers numerous advantages for the task at hand. Firstly, machine learning algorithms excel at handling complex datasets; this capacity allows for the analysis of various influencing factors such as land use, vegetation cover, and other geographical variables. These advanced approaches, such as neural networks [23], random forests [24], and support vector machines [25], can identify intricate patterns and relationships in datasets, leading to more accurate estimations of LST. Secondly, GIS technology enhances the visualization and geospatial analysis of LST data. By combining machine learning approaches, remote sensing data, and GIS, it is able to reveal insights into how different land cover types—such as green spaces, water bodies, and built-up areas—contribute to the SUHI effect [26,27,28].

## 2. Literature Review

In [25], a support vector machine (SVM) model was developed to predict LST; this study incorporated input parameters such as enhanced vegetation index, impervious surface density, and elevation. The model developed in [25] demonstrates the potential of the SVM model as a valuable tool for assessing the spatial variation of LST. Zhao et al. [29] relied on a random forest (RF) model to construct a non-linear relationship between LST and several critical factors, such as normalized difference vegetation index (NDVI), normalized difference water index (NDWI), and surface slope. The results demonstrated a high level of accuracy for the model, achieving a coefficient of determination (R^2^) exceeding 0.92. Khan et al. [30] investigated how the fluctuations in land use and land cover affected LST in planned and unplanned urban areas using Landsat data and machine learning techniques. The results reveal notable seasonal and annual differences in LST across these urban settings, with built-up areas showing the highest average LST, followed by bare soil and vegetation.

Lin et al. [31] also utilized RF regression to investigate the non-linear relationships between SUHI intensity and the morphological features of built-up areas. Their findings revealed that these morphological characteristics play a significant role in shaping urban thermal characteristics. Pande et al. [32] introduced a machine learning method for predicting LST that utilizes Landsat 8 satellite data in conjunction with advanced gradient boosting machines to support sustainable development. Their approach incorporates ensemble models and correlation analysis to estimate LST while exploring its functional relationships with other influencing factors. In this study, the Google Earth Engine was employed for data acquisition and processing. In [3], various machine learning algorithms, including Deep Neural Network (DNN) and gradient boosting algorithms, were used to assess the variation of LST across diverse land cover types such as built-up areas, soil, and vegetation. The study demonstrates the effectiveness of DNN and gradient boosting machines in predicting LST.

Ullah et al. [19] utilized SVM to evaluate the effects of land use and land cover changes on LST in Kabul, Afghanistan. The findings reveal a significant increase in LST corresponding with the expansion of built-up areas and a reduction in vegetation cover, highlighting a direct correlation between urbanization and intensified urban heat stress. Suthar et al. [20] relied on an artificial neural network (ANN) to predict LST in Bengaluru, India; this work demonstrated that ANN outperformed other models such as linear regression, SVM, and RF. The authors found that the prediction accuracy of the model varied by season, achieving an R^2^ of 0.92 for the data collected in summer and 0.95 for the data in winter. Mansourmoghaddam et al. [21] integrated a gradient boosting model for estimating LST and the Shapley Additive Explanation (SHAP) approach for assessing the impact of the explanatory variables.

Despite significant progress, there are still significant gaps in the research on spatial modeling of LST within urban environments. The studies that consider a comprehensive set of explanatory factors governing LST variations remain limited. For instance, in [33], building density was considered, but greenspace density was not taken into account. Tanoori et al. [3] employed land cover types and landscape metrics; however, the authors did not investigate the effect of spatial variables, such as distance to rivers and distance to coastlines. Furthermore, the recently proposed augmented normalized difference water index (ANDWI) and normalized difference bare soil index (NDBSI) have been shown to be effective in characterizing land surface properties in urban environments. However, these indices have rarely been used in urban LST modeling.

Although the trend of increasing LST in major urban areas is reported worldwide, further research is needed to identify the key variables that drive spatial variations in LST, given the distinct characteristics of each region. Notably, due to climate change, the significance of the explanatory variables for LST may fluctuate over time. Therefore, it is essential to examine the temporal evolution of these variables’ importance. Furthermore, given the complexity of the task at hand, there is a critical need for investigating the capability of other state-of-the-art machine learning approaches. Last but not least, although Da Nang’s urban center has recently experienced unprecedented heat waves and the intensifying SUHI effect due to rapid urbanization, the spatial variation of LST in Da Nang, Vietnam, has been rarely documented.

This study fills those gaps in the literature by proposing a data-driven approach for spatial modeling of LST in Da Nang’s urban center. Machine learning-based analyses are conducted to enhance the understanding of spatial variations of LST in the study area during the dry seasons of 2014, 2019, and 2024. To achieve the research goals, the current work investigates the effects of various environmental and anthropogenic factors on LST. Remote sensing data from Landsat 8 was utilized to retrieve LST and generate the GIS datasets. A set of topographical, spatial, and spectral index-based variables are employed as influencing factors. The Light Gradient Boosting Machine was employed to establish a functional relationship between LST and those influencing factors. To elucidate the factors contributing to LST variation, the Shapley Additive Explanations (SHAP) method is applied; the implementation of SHAP allows for the quantification of each factor’s impact and the identification of the most significant ones across different time periods. The proposed framework can be a helpful tool for urban planning authorities to estimate LST and implement strategies aimed at mitigating the effects of urban heat stress in Da Nang.

## 3. Materials and Methods

### 3.1. General Description of the Study Area

Da Nang, located in central Vietnam, is characterized by its diverse geographic features that include mountains, coastal plains, and Hoang Sa islands. Nestled between the East Sea to the east and the Truong Son Mountains to the west, the city has a unique topography with a wide range of elevation. The mountain range dominates the landscape, while the coastal region features beautiful sandy beaches and complex estuarine systems formed by rivers such as the Han and Cu De. Da Nang serves as a crucial transport hub, which connects neighboring urban centers and UNESCO World Heritage sites in Vietnam, such as Hue and Hoi An. The climate in the study area is characterized by its distinct wet and dry seasons.

**Figure 1:**
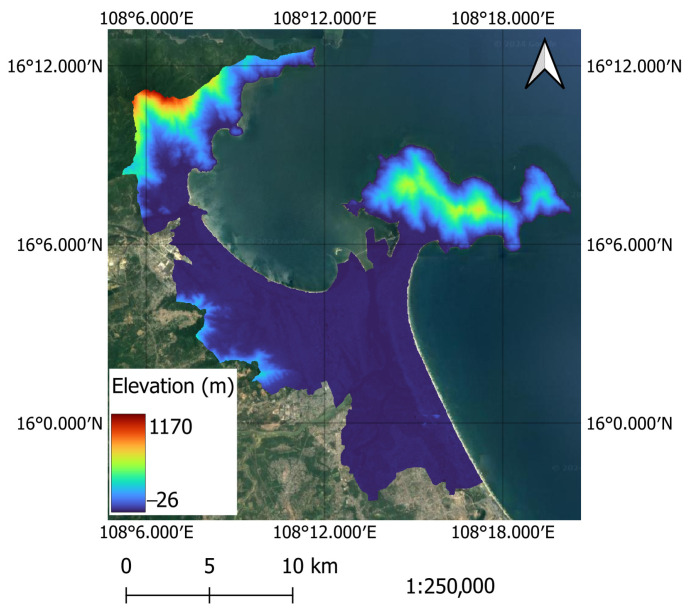
The study area.

The urban center of Da Nang is illustrated in Figure 1. This figure shows the location of the study area along with its elevation, ranging from −26 m to 1170 m. This region has experienced significant urban expansion. From 2002 to 2011, the city’s population grew by 1.3 times, reaching approximately 1,374,562 residents by 2021 [34]. Additionally, the urban area has rapidly increased, with built-up regions expanding by an average of 430.9 hectares each year between 1996 and 2015 [35]. Due to climate change and urbanization, urban areas in Da Nang are experiencing intensified heat stress. During the dry season, which usually spans from March to September, the difference in LST between the urban center and surrounding rural areas becomes considerable, intensifying the SUHI effect and creating significant heat discomfort within the urban environment. This SUHI effect leads to increased energy consumption for cooling, worsened air pollution, and greater health risks for city residents. Notably, during the dry season of 2024, Da Nang encountered an unprecedented heatwave, with a record-high air temperature reaching 40.7 °C on 26 April [36]. Therefore, there is a pressing need to employ advanced data-driven approaches for modeling LST in the study area, as well as to acquire insight into the factors influencing the spatial variation of this variable.

### 3.2. Remote Sensing Datasets

This study utilized data extracted from the Landsat 8 OLI/TIRS dataset to retrieve LST. This dataset, provided by the U.S. Geological Survey, is publicly accessible through the Google Earth Engine (GEE) code editor. The data cover the timeframe from 1 March to 30 September for the years 2014, 2019, and 2024. This dataset includes the thermal band (the 10th band) of the Thermal Infrared Sensor (TIRS) tier-1, which is utilized for measuring LST. It is noted that to enhance the image’s quality, the cloud masking method based on the Quality Assessment (QA) band was employed. Moreover, median filtering was applied to process the data acquired from the GEE data catalog.

The SR_5 and SR_6 bands of the Landsat 8 OLI/TIRS were utilized to compute the normalized difference vegetation index (NDVI) used in LST calculation. To construct the land cover maps and other spectral indices for the study area, the SR_2, SR_3, SR_4, SR_5, SR_6, and SR_7 bands of the Landsat 8 were used. Data regarding elevation of the study area were retrieved from the NASA SRTM Digital Elevation dataset [37] and processed with GEE. The general information of the used remote sensing datasets is summarized in Table 1. All thematic maps for the study area are prepared in QGIS.

### 3.3. Derivation of Land Surface Temperature (LST)

**Figure 2:**
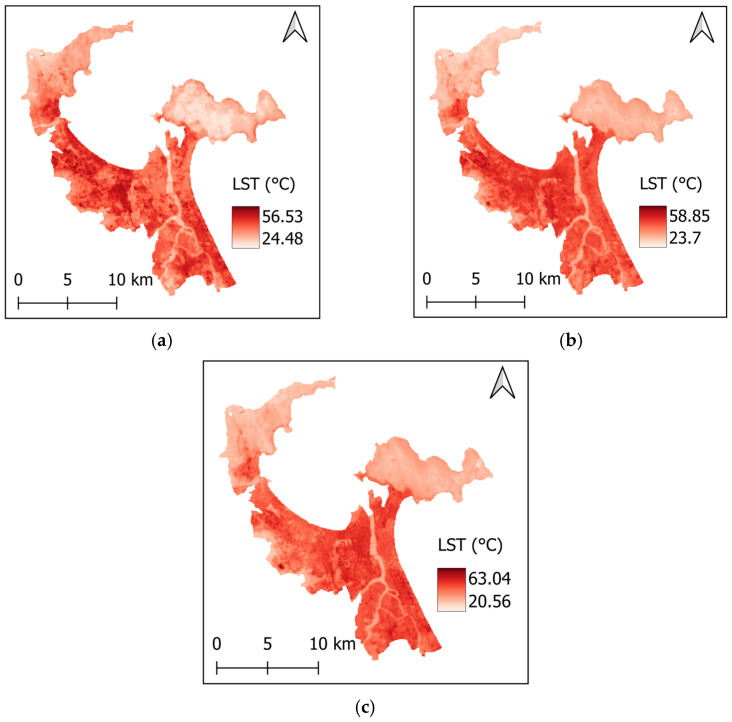
LST in the study area: (**a**) 2014, (**b**) 2019, and (**c**) 2024.

In this study, the data obtained from Landsat 8 OLI/TIRS sensors was filtered for the dry seasons (3 January–30 September) in 2014, 2019, and 2024. The resulting LST maps for the study area are prepared with QGIS (https://qgis.org/) and presented in Figure 2. To construct the LST maps, it is necessary to convert the spectral band values obtained from Landsat 8 into spectral radiance using a specific method [38] as follows:

(1) $$T_{S}=\frac{T_{B}}{1+(\lambda \times T_{B}/\rho )\times \ln(\varepsilon )}-273.15$$

where *B*_10_ denotes the digital number of the 10th band; *MRF* (0.0003342) and *ARF* (149) are the multiplicative rescaling and additive rescaling factors, respectively.

Moreover, the emissivity-corrected LST is computed in the following manner [39,40]:

(2) $$T_{S}=\frac{T_{B}}{1+(\lambda \times T_{B}/\rho )\times \ln(\varepsilon )}-273.15$$

where *T_S_* represents the estimated LST measured in Celsius (°C); λ (10.8 µm) denotes the wavelength of emitted radiance; ρ=h×c/b (1.438 × 10^−2^ mK), where *h* is the Planck’s constant (6.626 × 10^−34^ Js), *c* is the velocity of light (2.997 × 10^8^ m/s), and *b* is Boltzmann’s constant (1.38 × 10^−23^ J/K); the factor of 273.15 is used to convert the temperature from Kelvin (K) to Celsius (°C); the factor ε denotes the land surface emissivity.

The land surface emissivity (ε) is calculated as follows [41]:

(3) $$\varepsilon =0.004\times P_{\upsilon }+0.986$$

where $P_{\upsilon }$ denotes the vegetation proportion.

The vegetation proportion ($P_{\upsilon }$) is given by [38]:

(4) $$P_{\upsilon }=(\frac{NDVI-NDVI_{\min}}{NDVI_{\max}-NDVI_{\min}}{)}^{2}$$

where *NDVI*, *NDVI*_min_, and *NDVI*_max_ represent the value of NDVI, minimum NDVI, and maximum NDVI values at pixel level, respectively.

The 4th band (red) and 5th band (near infrared) of Landsat 8 are employed to compute the *NDVI* as follows:

(5) $$NDVI=\frac{NIR-R}{NIR+R}$$

where *NIR* and *R* are the bands of near infrared and red, respectively.

### 3.4. Explanatory Variables

**Figure 3:**
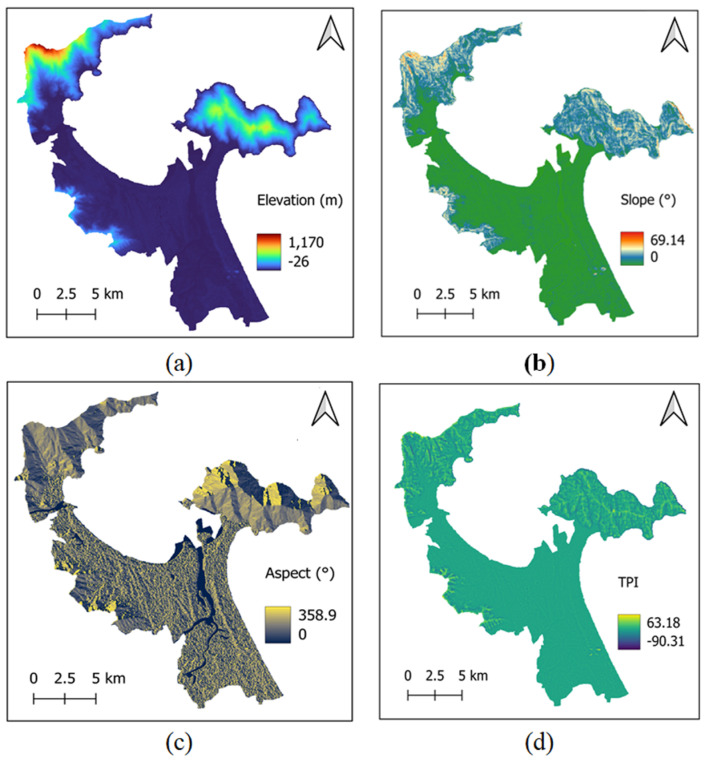
Topographic features: (**a**) elevation, (**b**) slope, (**c**) aspect, and (**d**) TPI.

Previous studies have demonstrated the strong effect of topographic variables on LST in urban environments [42]. Therefore, the current work relies on the variables of elevation, slope, aspect, and Topographic Position Index (TPI) to characterize the topographic features of Da Nang’s urban center. According to Phan et al. [43], elevation has a strong impact on the spatial distribution of LST. Meanwhile, slope plays a vital role in LST estimation due to its impact on solar radiation exposure; Peng et al. [42] demonstrated an apparent negative correlation between slope and LST. Aspect indicates the direction that a slope faces and therefore should be considered in LST estimation. TPI is used to assess the relative position of a point on the landscape compared to its surrounding terrain; this factor is helpful for landform classification [44] and therefore should be used for LST modeling. It is noted that slope, aspect, and TPI are computed via the GEE’s terrain module based on the elevation data provided in the NASA SRTM Digital Elevation dataset. The four topographic variables used in this study are presented in Figure 3.

**Figure 4:**
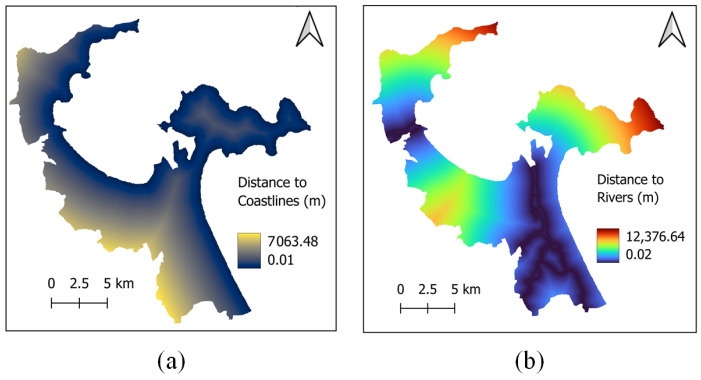
Spatial features: (**a**) distance to coastlines and (**b**) distance to rivers.

**Figure 5:**
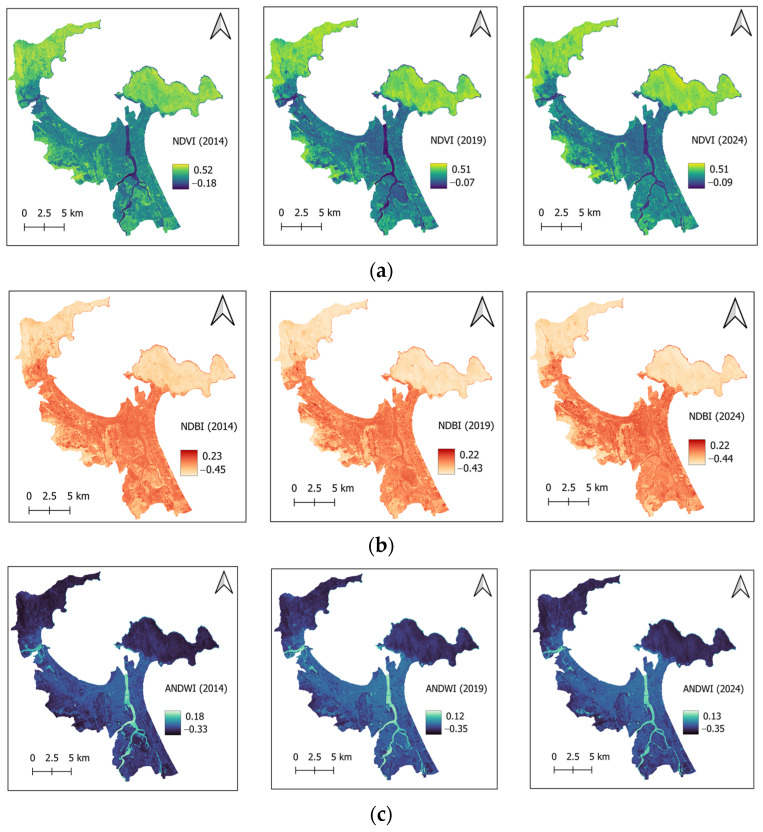

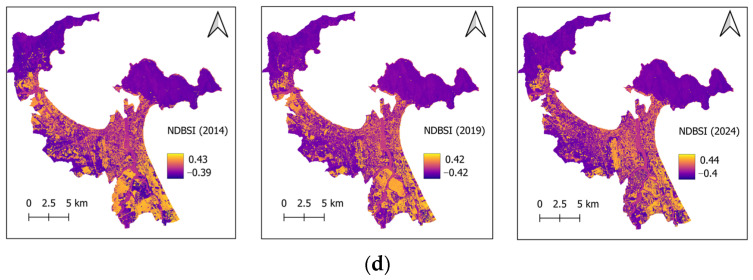
Spectral indices: (**a**) NDVI, (**b**) NDBI, (**c**) ANDWI, and (**d**) NDBSI.

Since water bodies provide substantial cooling effects in urban areas [45,46,47], the proximity of land to coastlines and rivers significantly influences LST. Therefore, the factors of distance to coastlines and distance to rivers (refer to Figure 4) are considered in the current work. Moreover, based on the findings in [48,49], NDVI (Figure 5a) and normalized difference built-up index (NDBI) (Figure 5b) are employed as explanatory variables for predicting LST. NDBI demonstrated a substantial positive correlation with LST; this index is computed as a normalized difference between the shortwave infrared and near infrared bands [49].

NDBI is an important metric used to identify and quantify built-up areas within a landscape. The values of NDBI range from −1 to 1, where positive values typically indicate the presence of built-up surfaces, such as buildings and roads, while negative values are associated with vegetation and water bodies. NDVI, ranging from −1 to 1, is a widely used metric for assessing vegetation health and density. Negative values of NDVI typically indicate non-vegetated surfaces. An NDVI value of zero generally corresponds to bare soil or areas with little to no vegetation. For positive NDVI values, higher numbers indicate healthier vegetation in the corresponding area. In addition, there is a commonly inverse relationship between LST and NDVI due to the cooling effect of vegetation [48].

Besides these two commonly used spectral indices, the recently proposed augmented normalized difference water index (ANDWI) [50] and normalized difference bare soil index (NDBSI) [51] are employed in this study to characterize the properties of land surface. The thematic maps of ANDWI and NDBSI are presented in Figure 5c,d, respectively.

*ANDWI* was demonstrated as a robust water index useful for delineating water from non-water areas; this index is computed as follows [50]:

(6) $$ANDWI=\frac{B+G+R-NIR-SWIR_{1}-SWIR_{2}}{B+G+R+NIR+SWIR_{1}+SWIR_{2}}$$

where *B*, *G*, *R*, *NIR*, *SWIR*_1_, and *SWIR*_2_ are the bands of blue, green, red, near infrared, shortwave infrared 1, and shortwave infrared 2, respectively.

ANDWI is formulated in [50] to enhance the detection of water bodies by maximizing the contrast between water and non-water pixels using a combination of spectral bands. The range of ANDWI values typically spans from −1 to 1. Positive ANDWI values generally indicate the presence of water bodies. As the ANDWI value increases towards 1, it signifies a higher concentration of water. Zeros and negative ANDWI values are generally associated with transition zone and non-water surfaces, respectively.

Moreover, *NDBSI* is effective for mapping bare soil in urban areas; this spectral index is calculated as follows [51]:

(7) $$NDBSI=\left\{\begin{array}{l} -\frac{\left|SWIR_{1}-B\right|}{\left|SWIR_{1}+B\right|}\times k,\;k<0\\ \frac{SWIR_{1}-B}{SWIR_{1}+B}\times k,\;k>0 \end{array}\right.$$

where

(8) k=r×v

(9) $$r=1-\frac{SWIR_{1}-NIR}{3\times |NIR-R|}$$

(10) v=R−G

NDBSI ranges from −1 to 1. Positive values indicate the presence of bare soil, with higher values suggesting a greater concentration of this land cover type. A value of zero typically corresponds to water bodies, while negative values are associated with vegetation and impervious surfaces. This index is particularly useful for land cover classification and the identification of bare soil in urban environments [51].

### 3.5. Machine Learning Approaches

#### 3.5.1. Light Gradient Boosting Machine (LightGBM)

**Figure 6:**
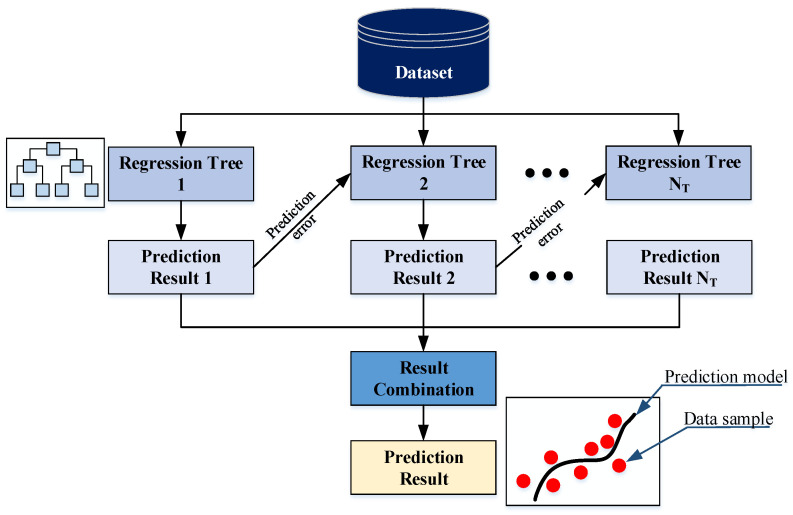
LightGBM prediction model.

The Light Gradient Boosting Machine (LightGBM), introduced in [52], is an advanced gradient boosting framework that utilizes decision trees as its base models. This machine learning technique aggregates multiple weak regressors to form a robust model. The construction of a LightGBM model occurs sequentially; each iteration aims at reducing the prediction error from the previous model. The model structure is demonstrated in Figure 6.

The ensemble model *f*(*x*) is created by merging a collection of *M* decision trees as follows:

(11) $$f(x)=\sum_{m=1}^{T}f_{m}(x)$$

where *f*_1_, *f*_2_, …, *f_T_* are the base models in the ensemble.

Notably, the LightGBM model employs a leaf-wise algorithm to grow trees in a vertical manner. It selects the leaf that provides the greatest reduction in the loss function for splitting and expanding the decision tree. Additionally, the training efficiency of LightGBM is improved by the Gradient-based One-Side Sampling (GOSS). GOSS directs the model’s attention toward samples with larger gradients. This approach enables LightGBM to focus on more informative data points during the learning process. Furthermore, the model utilizes Exclusive Feature Bundling (EFB) that combines mutually exclusive features to achieve automatic feature reduction and retention of the most informative explanatory variables [53].

#### 3.5.2. Support Vector Machine (SVM)

Support vector machine (SVM) for regression analysis [54] is an adaptation of the original support vector machine that incorporates ε-insensitive hinge loss. This particular loss function establishes a margin of tolerance, meaning that prediction errors within this range are not penalized. SVM is considered a nonparametric model because it relies on kernel functions during its inference process. The training phase of this model focuses on identifying a hyperplane *f*(*x*) that minimizes structural risk in the feature space. Due to the employment of insensitive hinge loss and the principle of structural risk minimization, SVM typically exhibits strong generalization capabilities and has been effectively utilized for various learning tasks [19,25]

#### 3.5.3. Random Forest (RF)

The random forest (RF) algorithm, proposed in [55], is a flexible ensemble learning technique suitable for nonlinear function approximations. This machine learning method creates an ensemble of decision trees during the training process, which then provides estimated values for regression (i.e., LST). RF harnesses the strengths of multiple weak learners to improve model accuracy and robustness, thereby minimizing the risk of overfitting commonly associated with individual decision trees. Each tree in the forest is trained on a randomly selected subset of the data, with features also chosen randomly at each split. This property helps enhance the diversity within the model. This aggregate of varied results from multiple trees enables RF to effectively handle complex datasets characterized by high dimensionality and nonlinear relationships.

#### 3.5.4. Deep Neural Network (DNN)

Deep Neural Network (DNN) is a powerful tool for regression analysis. This model consists of a series of hidden layers, where each layer receives input from the output of the previous layer [56]. Unlike traditional regression methods, which often rely on linear assumptions, DNNs can capture intricate nonlinear patterns through their layered architecture. This flexibility makes them particularly effective for applications where relationships between input features and target variables are highly nonlinear. Each layer in a DNN model includes interconnected neurons that process input data through mathematical operations. The hidden layers play a crucial role in transforming the raw input into abstract representations, allowing the network to learn from the data in a hierarchical manner. This capability enables DNN to handle high-dimensional datasets and uncover complex patterns.

### 3.6. Shapley Additive Explanations (SHAP)

Shapley Additive Explanations (SHAP) [57] is an advanced framework designed to enhance the interpretability of machine learning models, particularly in regression analysis. Inspired by cooperative game theory, SHAP provides a systematic approach to quantifying the contribution of each feature to a model’s predictions. This transparency is crucial in regression tasks, where understanding how individual predictors influence outcomes can significantly impact decision making. The primary advantage of SHAP lies in its ability to generate consistent and objective explanations for model predictions. By calculating SHAP values, analysts can determine the extent to which each feature affects the predicted outcome. Positive SHAP values indicate a feature’s contribution to increasing the prediction, while negative values signify a reduction. This detailed insight allows for the identification of influential features.

SHAP is a valuable tool for analyzing machine learning-based models for estimating LST [58,59]. By providing insights into how individual features contribute to model predictions, SHAP enhances the interpretability of complex regression models often used in environmental studies. One of the key advantages of SHAP is its ability to quantify the impact of various factors on LST. By calculating SHAP values, it is able to identify which features are most influential in determining LST and understand their respective contributions. This capability is particularly beneficial when dealing with intricate datasets that include multiple interacting variables.

### 3.7. Metrics for Model Performance Evaluation

In the context of spatial modeling of LST, the task is formulated as a function approximation problem, where the goal is to accurately predict temperature in urban areas values based on various explanatory variables. To achieve this goal, the current work employs advanced machine learning algorithms, including LightGBM, SVM, RF, and DNN. Evaluating the performance of these models is crucial to understanding their effectiveness and suitability for the task at hand. To this end, multiple metrics are used; they include Root Mean Square Error (*RMSE*), Mean Absolute Percentage Error (*MAPE*), Mean Absolute Error (*MAE*), and coefficient of determination (*R*^2^). Each of these metrics offers unique insights into model performance. Moreover, using a combination of metrics provides a more comprehensive evaluation than relying on a single measure.

The aforementioned metrics are computed as follows:

(12) $$RMSE=\sqrt{\frac{1}{N}\sum_{i=1}^{N}{\left(y_{i}-t_{i}\right)}^{2}}$$

(13) $$MAE=\frac{1}{N}\times \sum_{i=1}^{N}\left|y_{i}-t_{i}\right|$$

(14) $$MAPE=\frac{100}{N}\times \sum_{i=1}^{N}\frac{\left|y_{i}-t_{i}\right|}{y_{i}}$$

(15) $$R^{2}=1-\frac{\sum_{i=1}^{N}{\left(t_{i}-y_{i}\right)}^{2}}{\sum_{i=1}^{N}{\left(t_{i}-\bar{t}\right)}^{2}}$$

where *t_i_* and *y_i_* denote the actual and estimated LST values of the *i*th sample, respectively. *N* represents the total number of samples in the dataset.

## 4. The Proposed Machine Learning Framework for LST Estimation

**Figure 7:**
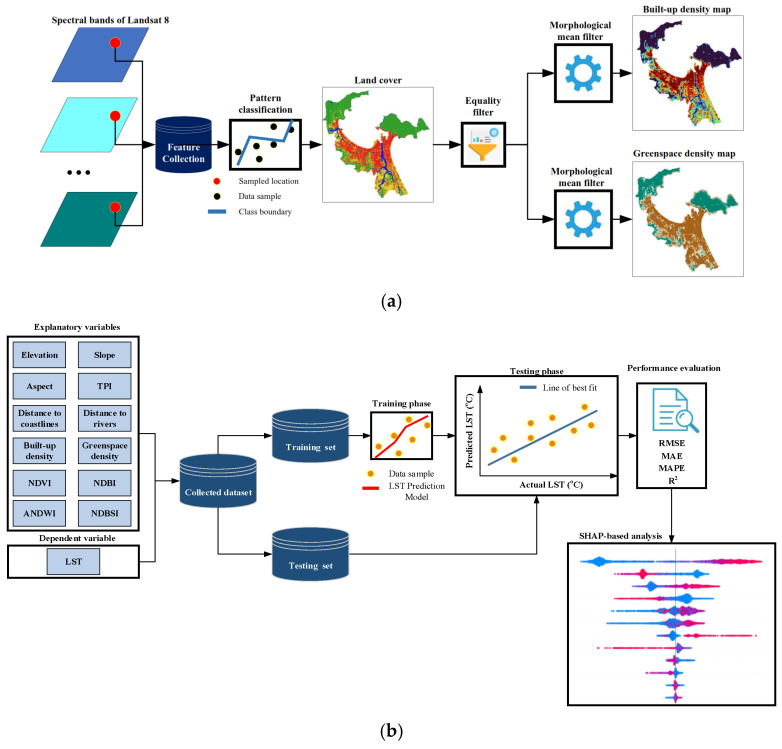
The proposed framework: (**a**) density maps and (**b**) LST modeling.

This research proposes an integrated framework (refer to Figure 7) for estimating the spatial variation of LST using machine learning methods, geospatial analysis, and remote sensing data. The variables of elevation, slope, aspect, TPI, distance to coastlines, distance to rivers, built-up density, greenspace density, NDVI, NDBI, ANDWI, and NDBSI are used as LST’s influencing factors. To compute built-up density and greenspace density, the spectral bands of Landsat 8 are employed as input features for random forest classifiers to construct land cover maps for the study area in 2014, 2019, and 2024. This pattern recognition phase is performed in GEE with its classifier module. Based on the classified land cover maps, equality filters and morphological mean filters (with a radius of three pixels) are used to estimate the maps of built-up density and greenspace density (as shown in Figure 7a).

The built-up density and greenspace density both range from 0 to 1. These two indices quantify the proportion of urbanized land relative to the total area and the extent of vegetation cover, respectively. Built-up density indicates the intensity of development within a given area, reflecting how much land is occupied by buildings and infrastructure. In contrast, greenspace density measures the availability of natural areas, such as parks and forests, contributing to the enhancement of urban heat comfort.

The proposed framework for spatial modeling of LST in Da Nang, Vietnam (refer to Figure 7b) utilizes LightGBM to effectively generalize the relationship between LST and twelve independent variables. This framework integrates a variety of data sources along with advanced computational techniques and is capable of providing a powerful tool for analyzing and predicting variations in urban LST. To validate the predictive performance of LightGBM, this study compares its accuracy against other algorithms, including RF, SVM, and DNN. The objective of this comparison is to determine which algorithm yields the highest accuracy in mapping LST for the study area.

The LightGBM model is implemented via the toolbox provided in [60]. The RF, SVM, and DNN models are built with the assistance of the Scikit-learn library [61]. All hyper-parameters of the machine learning models are specified by the cross-validation processes [62]. The models are built and executed in Microsoft’s Visual Studio for Python programming (https://visualstudio.microsoft.com/vs/features/python 3.10/, accessed on 30 July 2023). To quantify the prediction performance, the four metrics of RMSE, MAE, MAPE, and R^2^ are employed. Furthermore, the framework incorporates SHAP to evaluate the impact of various features on LST estimation. By computing SHAP values, the analysis provides valuable insights into the significance of each explanatory variable influencing LST. This application of SHAP enhances the interpretability of the model’s predictions; the analysis outcome allows for a clear understanding of the factors’ criticality in each time period.

## 5. Results

### 5.1. Land Cover and Density Mapping

Because impervious surfaces and greenspace significantly impact LST [46,63,64], this study utilizes the density of built-up areas and greenspaces as independent variables. To compute these variables, the land cover maps of the study area in 2014, 2019, and 2024 are constructed by the spectral bands of Landsat 8 and the random forest classifiers in GEE. Herein, this classifier is trained to categorize the pixels in the study area into four distinctive classes: built-up, vegetation, barren, and water.

The land cover classification process utilizes a dataset comprising 400 samples for each year, with each class containing 100 samples. The ground truth labels for these samples have been verified through both Google Earth Pro and field visits. For each year, the dataset is randomly divided into a training set (70%) and a testing set (30%). The training set is employed to train the random forest classifiers within GEE, while the testing set is used to evaluate the model’s generalization capability.

**Figure 8:**
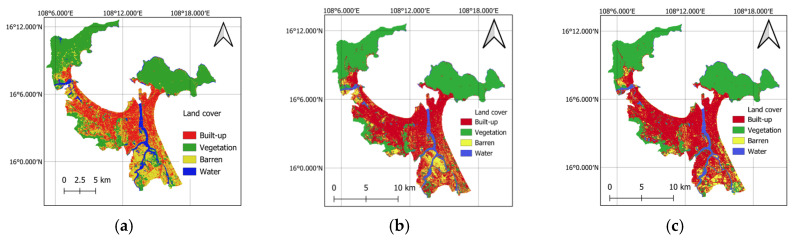
Maps of land covers: (**a**) 2014, (**b**) 2019, and (**c**) 2024.

**Figure 9:**
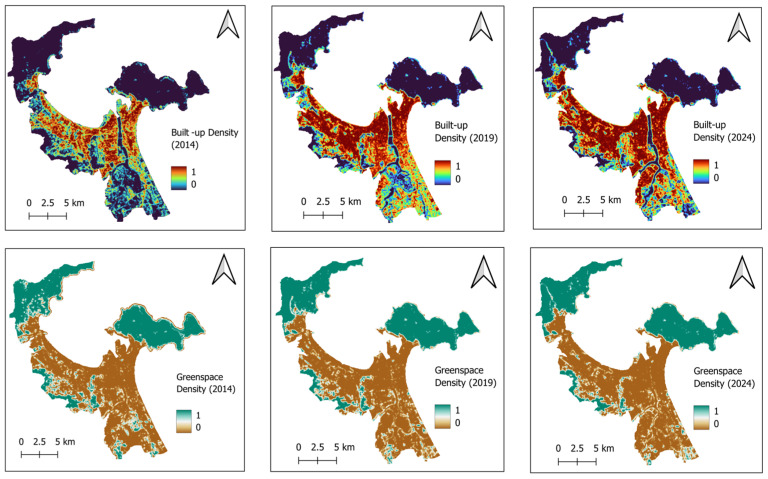
Maps of built-up and greenspace density.

The random forest classifier is used in this section because this machine learning ensemble has demonstrated superior performance in land cover mapping [65,66]. Moreover, this classification model can be implemented easily through the built-in functions provided in GEE [67,68]. The classifier’s performance in GEE can also be optimized without sophisticated programming expertise. Based on experimental results, the random forest classifiers generally demonstrate robust performance; they achieve classification accuracy rates of 0.91, 0.93, and 0.91 for the years 2014, 2019, and 2024, respectively. These results reflect the classifiers’ effectiveness in accurately distinguishing between different land cover types. The resulting land cover maps generated from this classification process are illustrated in Figure 8, which shows the spatial distribution of various land cover classes over the specified years. Based on the land cover maps, equality filters are used to identify pixels associated with the built-up and greenspace classes. Subsequently, the maps of urban built-up density and greenspace density can be obtained via the morphological mean filters; these maps are presented in Figure 9.

### 5.2. Spatial Modeling of LST and Prediction Result Comparison

Based on the aforementioned density maps and other influencing factors, the LightGBM machine learning approach is used to construct regression models for LST estimation in Da Nang’s urban center. Furthermore, to develop the model for estimating LST, 3000 data points were randomly sampled from the study area. This dataset is subsequently split into two parts: a training set and a testing set. The training set comprised 70% of the total samples, while the remaining 30% was used as the testing set.

**Figure 10:**
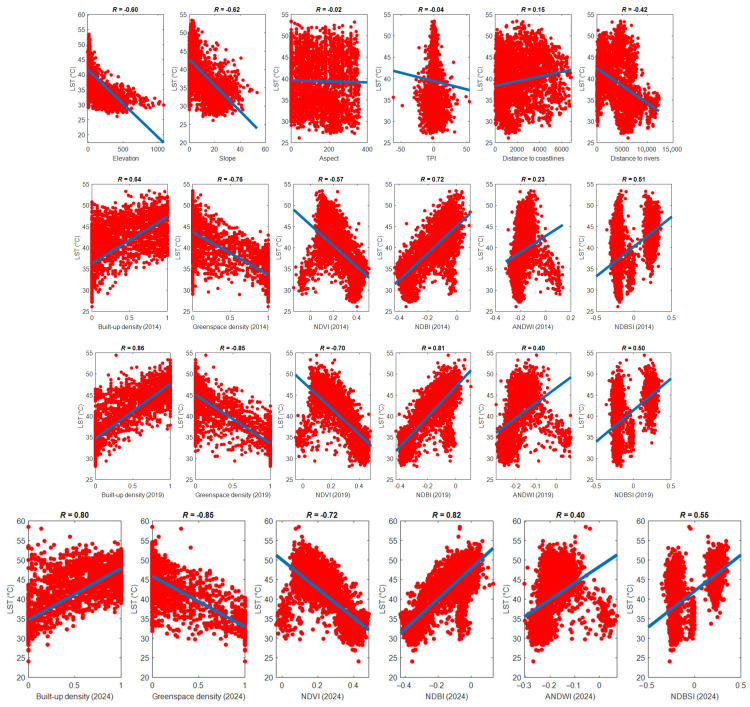
Correlations between the independent variables and LST.

Moreover, the correlations between the influencing factors and LST are demonstrated in Figure 10. Herein, the Pearson correlation coefficient (R) is calculated to assess the linear relationships between LST and each explanatory variable. The results of the Pearson correlation analysis demonstrate significant linear associations between various land use and environmental factors and LST in the study area. In 2014, the strongest negative correlation was found between greenspace density and LST, with an R value of −0.76. This suggests that increased greenspace density is associated with lower temperatures, highlighting the cooling effect of vegetation in Da Nang’s urban areas. A similar trend was observed in 2024, where the correlation remained strong at −0.85, further emphasizing the importance of greenspaces in mitigating urban heat.

In 2019, the strongest positive correlation was found between built-up density and LST, with an R of 0.86. This indicates that areas with higher built-up density tend to experience elevated temperatures; this fact accentuated the effect of urbanization in the study area during the time period of interest. Additionally, NDBI demonstrated a strong influence on LST, with R values ranging from 0.72 to 0.82; this fact confirmed the relationship between urbanized areas and increased temperatures. NDVI exhibited a moderate to strong negative correlation with LST, with R values between −0.57 and −0.72. This highlights the cooling effects of vegetation. Moreover, NDBSI displayed a moderate positive correlation with LST, with R values from 0.50 to 0.55, indicating that regions characterized by exposed soil or sparse vegetation are linked to higher temperatures.

ANDWI showed a relatively weak correlation with LST, with R values ranging from 0.23 to 0.40; the scatter plots in Figure 10 suggested a nonlinear relationship between ANDWI and LST. Additionally, a positive correlation was observed between distance to coastlines and LST (R = 0.15), implying that areas closer to coastlines may experience slightly lower temperatures. For the topographical features, elevation and slope exhibited moderate linear correlations with LST, while aspect and TPI showed weaker correlations.

The results from the LightGBM model for estimating LST demonstrate a strong performance across various metrics, as summarized in Table 2. The RSME indices of the model are 2.30, 1.64, and 2.02 for the 2014, 2019, and 2024 time periods, respectively. RMSE quantifies the difference between predicted values and actual observations. However, since RMSE squares the errors before averaging, larger discrepancies have a disproportionately significant impact on the final value. Therefore, this metric is relatively sensitive to outliers. In addition, MAE measures the average magnitude of errors in predictions, treating all discrepancies equally without considering their direction. Hence, MAE is robust against outliers. The model achieves MAEs ranging from 1.22 to 1.77. This fact implies that on average, the model’s predictions deviate from the actual values by a range of 1.22 to 1.77 °C. This range indicates that while the model performs reasonably well in terms of accuracy, there is variability in its predictive capability across different time periods.

**Figure 11:**
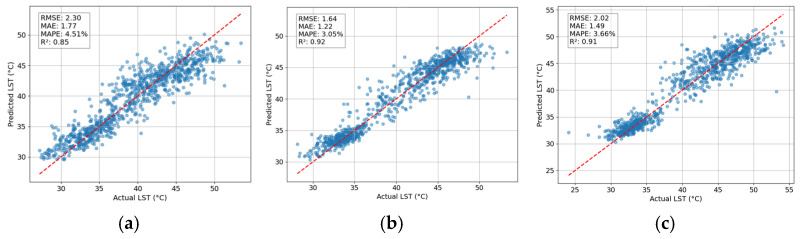
LightGBM prediction results: (**a**) LST in 2014, (**b**) LST in 2019, and (**c**) LST in 2024.

The R^2^ values indicated strong performance of LightGBM. R^2^ was recorded at 0.85 for 2014, improving to 0.92 in 2019 and slightly decreasing to 0.91 by 2024. The experimental results show that the model is capable of explaining 85% of the variation in the LST for the data in 2014. In 2019 and 2024, the model can capture more than 90% of the variation in the LST. The prediction results of LightGBM are further displayed in the scatter plots provided in Figure 11. Herein, the red dashed line is the line of best fit, indicating a perfect agreement between actual and estimation LST. Each point in the scatter plot shows a prediction outcome of the model. The closer the point is to the line of best fit, the more accurate the prediction.

**Figure 12:**
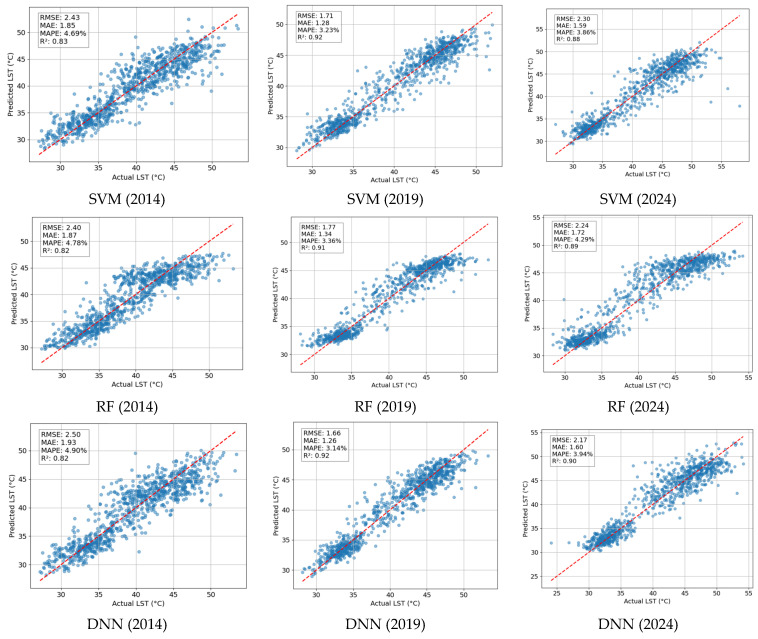
Prediction results of benchmark models.

Moreover, the performance of the benchmark machine learning approaches is summarized in Table 3. The LightGBM model achieved the lowest RMSE for all datasets. In 2014, RF was the second-best model with an RMSE of 2.40, followed by SVM (RMSE = 2.43) and DNN (2.50). In 2019 and 2024, the performance of DNN (RMSE = 1.60 for 2019 and RMSE = 2.17 for 2024) is only slightly inferior to LightGBM. The prediction outcomes of the benchmark models are further displayed in Figure 12. Based on the results in this figure, LightGBM clearly outperforms other benchmark methods for the datasets from 2014 and 2024. However, in terms of R2, LightGBM, SVM, and DNN achieve comparable performance for the dataset from 2019. These outcomes are reasonable since SVM, RF, and DNN were demonstrated to be capable models for geospatial data analysis [69,70,71].

### 5.3. Analysis of Independent Variables Based on SHAP

Sensitivity analysis is crucial in understanding the impact of various features on LST, especially in studies addressing SUHI in the context of rapid urbanization and climate change. SHAP analyses allow for quantifying the contribution of each feature to the model’s predictions, providing insights into how different environmental and urban factors influence LST [26]. In this study, since LightGBM has gained the most desired performance in spatial modeling of LST, this method is used along with SHAP to offer an interpretable ranking of feature importance, enabling urban planners to identify which factors most significantly affect LST in Da Nang’s urban area from 2014 to 2024. It is noted that LightGBM leverages TreeSHAP for computing SHAP values, which allows for rapid calculation of feature contributions without the need for extensive sampling [72,73]. This fact increases the computational efficiency of the sensitivity analysis.

SHAP analyses can be graphically presented as impact plots. These plots are powerful visualization tools used to interpret the contributions of individual variables to the predictions of LightGBM. These plots leverage concepts from cooperative game theory, specifically Shapley values, to provide insights into how different input variables influence the output of machine learning models. Herein, SHAP values quantify the contribution of each feature to the prediction of LST for a specific data point. Each explanatory variable is treated as a “player” in a game, where the goal is to determine how much each player contributes to the total payout (i.e., the LST prediction). The SHAP value for a feature indicates its average contribution across all possible combinations of features, allowing for both local (individual prediction) and global (overall model behavior) interpretability.

**Figure 13:**
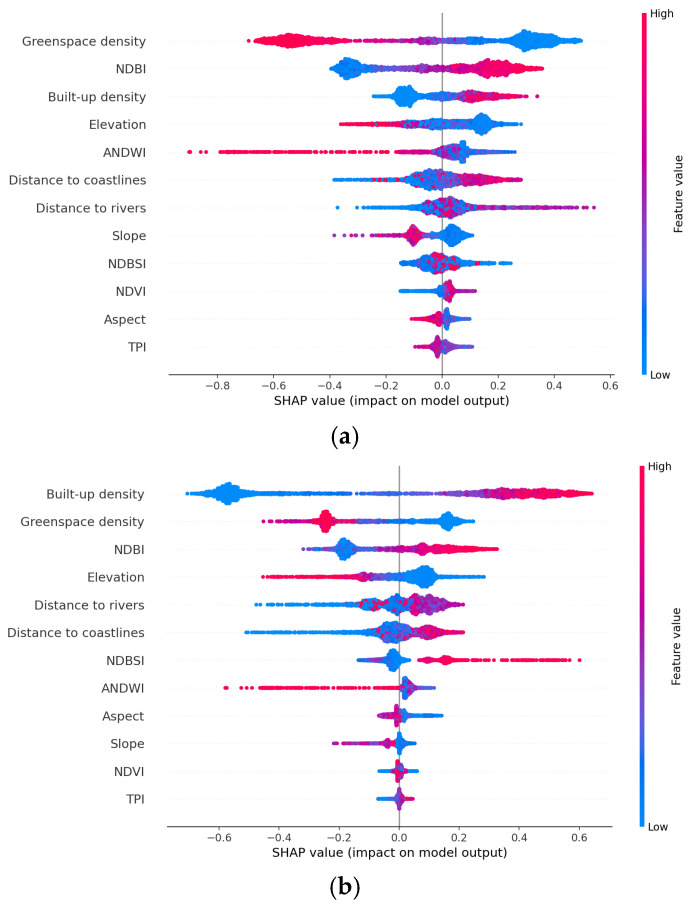

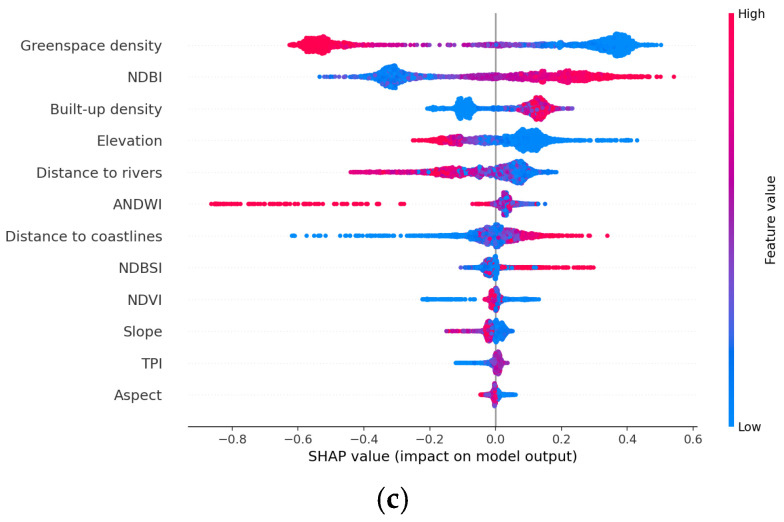
SHAP impact plots: (**a**) 2014, (**b**) 2019, and (**c**) 2024.

The impact plots yielded by SHAP for the datasets in 2014, 2019, and 2024 are provided in Figure 13. Generally, there are clear temporal changes in feature ranking. The analysis illustrates shifting priorities among influential features across different years:

(i) In 2014, greenspace density ranked first, followed by NDBI, built-up density, elevation, and ANDWI.

(ii) In 2019, built-up density was the most influential variable, followed by greenspace density and NDBI.

(iii) In 2024, greenspace density regained prominence, indicating its crucial role in affecting LST variation in the study area.

Both built-up density and greenspace density consistently appear among the top three influential factors. This duality highlights how urbanization can intensify the heat stress while simultaneously emphasizing the cooling benefits of greenspaces in Da Nang’s urban center. Elevation is noted as a significant factor impacting LST, suggesting that topographical variations in the study area can influence temperature distributions. However, other topographical features like slope and aspect have lesser effects compared to elevation. NDBI and NDBSI also show significant positive correlations with LST; this finding emphasizes the influence of urbanization and infrastructure on elevated temperatures. Furthermore, negative correlations observed with greenspace density, ANDWI, distance to rivers, elevation, and slope suggest these features contribute to cooling effects. This finding emphasizes the importance of integrating natural landscapes into urban planning to mitigate the heat stress.

## 6. Discussion

### 6.1. Changes in Land Cover Classes

Based on the results reported in the previous section, it can be seen that the changes in land cover classes over the years in the study area have a notable relationship with the variations in LST. Analyzing the data from 2014, 2019, and 2024 (refer to Table 4) reveals important trends that connect urbanization and temperature increases. From 2014 to 2024, there has been a considerable increase in built-up areas, which expanded from approximately 67.97 km^2^ to 106.51 km^2^. This increase in built-up density correlates with rising mean LST values, which rose from 39.38 °C in 2014 to 40.49 °C in 2024. The maximum LST also increased significantly during this period, from 56.53 °C to 63.04 °C. These trends suggest a direct link between the expansion of urban infrastructure and rising temperatures in Da Nang’s urban center.

Conversely, vegetation cover has decreased slightly from 102.83 km^2^ in 2014 to 98.47 km^2^ in 2024. This reduction in vegetation is critical because vegetation plays a vital role in cooling through evapotranspiration and shading effects. This decline in vegetative cover might contribute to the observed increases in LST, as less greenspace means less natural cooling. Moreover, the barren area has decreased dramatically from 55.33 km^2^ in 2014 to just 22.64 km^2^ by 2024. While this might seem beneficial for urban development, it further reduces natural land cover. A large portion of barren areas have been replaced by impervious surfaces (e.g., asphalt and concrete), which absorb and retain heat more than natural surfaces do. The area of water bodies shows a slight decrease from 13.64 km^2^ to 11.21 km^2^ but then increased slightly to 12.16 km^2^ by 2024. Water bodies can help moderate temperatures through their thermal properties; however, their limited presence compared to built-up areas means they cannot sufficiently offset the rising temperatures caused by the expansion of impervious surfaces.

### 6.2. Effect of Land Cover Changes on the Accuracy of LST Modeling

In the task of LST estimation, LightGBM has demonstrated superior performance compared to RF, SVM, and DNN. This finding highlights several advantages of LightGBM that contribute to its effectiveness in this context. First, LightGBM excels at capturing complex, non-linear relationships between variables, which is essential for accurately modeling LST influenced by various factors such as land cover, elevation, and urbanization.

In particular, LightGBM has several benefits for regression analysis tasks, including capabilities for parallel training, regularization, and optimization for sparse data. As reviewed in [74], the primary advantage of LightGBM lies in its advanced tree construction method. LightGBM employs a leaf-wise splitting approach, which selects the node with the greatest delta loss after each split for subsequent splits. This strategy allows LightGBM to efficiently process large datasets. Additionally, it relies on a histogram-based technique to determine the optimal split, which significantly reduces training time.

This capability allows it to outperform traditional models like RF and SVM that may not fully capture the intricate patterns in the datasets. Second, LightGBM incorporates advanced regularization techniques to mitigate overfitting; this capability ensures that the model generalizes well to unseen data. This finding is in line with the previous works [73,75,76] that accentuated the predictive capability of LightGBM.

In comparing the predictive performance of the current study with the ones reported in recent works, the current work demonstrates competitive accuracy with R^2^ of 0.85, 0.92, and 0.91 in 2024, 2019, and 2024, respectively. In [64], DNN and RF both achieved R^2^ values of 0.83, while an extreme gradient boosting machine (XGBoost) model reached 0.85. The XGBoost model in [26] attained an R^2^ of 0.87, which is still below the best performance level achieved in the current work. These facts highlight the effectiveness of LightGBM in accurately estimating the spatial variation of LST in the study area.

The ability of LightGBM in LST estimation can be helpful for developing climate adaptation strategies, particularly in regions experiencing extreme heat events. Policymakers can utilize these models to identify vulnerable areas and implement cooling measures effectively. The findings encourage further exploration of LightGBM’s capabilities in other contexts, such as simulation and mitigation of severe heat stress [77], spatial modeling of ozone and SUHI [78], and forecast of the effect of urbanization on LST [79].

**Figure 14:**
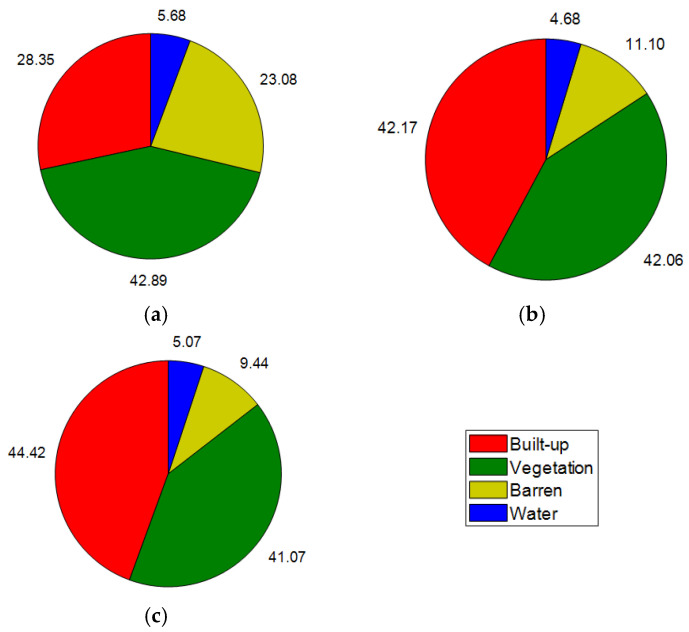
Proportions of land cover in each year: (**a**) 2014, (**b**) 2019, and (**c**) 2024.

The model performance in 2019 and 2024 (with an R^2^ of more than 0.90) is better than that in 2014 (with an R^2^ of only 0.85). From the view of proportions of land cover in each year (refer to Figure 14), it can be seen that the proportion of built-up areas increased significantly from 28.35% in 2014 to 42.17% in 2019, and subsequently slightly increased to 44.42% in 2024. The proportion of barren areas reduced drastically from 23.08 to 11.10 in 2019 and further reduced to 9.44% in 2024. There are no significant changes in the proportions of vegetation and water bodies.

The differences in prediction performance for LST in urban areas across the years 2014, 2019, and 2024 can be analyzed through the lens of fractal dimension (FD) for the land cover types. FD is a measure of complexity and spatial distribution of land cover. Higher FD values typically indicate more complex and heterogeneous landscapes, while lower values suggest more uniform or simpler patterns. This study employs the box-counting method to compute the FD of land cover categories. The box-counting method has been widely used for measuring the fractal dimension of spatial structures in previous studies [80,81]. This method provides insights into the complexity and self-similar characteristics of land cover types.

The box-counting method involves overlaying a grid of boxes of varying sizes onto an image of the land cover type being analyzed. The process begins by counting the number of boxes that contain any part of the land cover pattern, regardless of whether they are fully filled or only partially occupied. This count is recorded as a function of the size of the boxes used. The relationship between the number of non-empty boxes and the size of these boxes is then analyzed statistically to derive the fractal dimension.

A linear regression analysis is performed on the data for each land cover type. The slope of this linear equation is used to represent FD. This index provides a quantitative measure of the complexity of the spatial arrangement of land cover types. FD varies from 1 to 2 with 1 indicating a simple, linear structure often associated with uniform land covers and 2 suggesting a more complex and filled structure typical of built-up regions, where intricate patterns emerge due to various urban elements such as buildings and roads. FD values between 1 and 2 reflect mixed characteristics, indicating varying degrees of complexity in land cover types. In this study, the box-counting method is implemented in Python 3.10 with the assistance of the Rasterio Python toolbox [82]. The analysis results are summarized in Table 5.

Notably, the increasing FD values for built-up areas indicate a growing complexity in their spatial arrangement over time. This trend suggests that as urban development progresses, built environments become more intricate, characterized by a mix of structures and layouts that contribute to higher heat retention and SUHI effects. The significant decrease in FD for barren areas indicates a loss of complexity in their spatial arrangement over time. This decline may be attributed to urban encroachment that simplifies the landscape, leading to more uniform and less varied barren regions. The increasing complexity of built-up areas highlights the ongoing urbanization process in the study region. This is consistent with the information presented in Table 5. The reduction in complexity within barren areas points to a significant conversion of this land cover type into built environments.

As reported in Table 5, the FD for built-up areas increased from 1.6239 in 2014 to 1.6889 in 2024; this outcome indicates urban growth and increased complexity in land use patterns over time. As urbanization progresses, the increased complexity (higher FD) allows for more nuanced interactions between different land cover types, which can improve model predictions of LST due to more diverse thermal responses from various surfaces. This expansion in built-up areas (67.97 km^2^ in 2014 to 106.51 km^2^ in 2024) likely leads to increased complexity in land cover patterns, which can enhance the model’s ability to predict LST accurately. Furthermore, the reduction in barren land may lead to less variability in thermal properties, as barren surfaces often have different and inconsistent heat retention and radiation characteristics compared to developed or vegetated surfaces.

In 2014, with a smaller built-up area (67.97 km^2^) and a higher proportion of barren land (23.08%), the landscape was less complex in terms of thermal interactions. This fact may create more difficulty for the model to capture the LST variations accurately. The presence of more barren land could lead to greater uncertainty in LST predictions. Therefore, the prediction model is only capable of explaining 85% of the variation in LST. In 2019 and 2024, the information contained in data points where the city expands provides more variability of the LST to the machine learning model. This fact allows LightGBM to better generalize about urban heat dynamics and improve prediction accuracy (R^2^ = 0.92 in 2019 and R^2^ = 0.91 in 2024).

### 6.3. SHAP Insights into the Impact of Explanatory Variables

The SHAP-based analysis reveals that built-up density, greenspace density, and NDBI significantly influence LST variations. These findings align with previous studies of [4,83], which confirmed the intensified heat stress due to reduced green areas and expansion of built-up areas. As pointed out in [84], the expansion of built-up areas is a primary driver of observed LST changes. The impact of bare soil on LST variation remains ambiguous. Peng et al. [85] reported a positive correlation between bare soil and LST, while Chen et al. [86] suggested a negative relationship. This ambiguity is explained by the complexity of urban land cover interactions and their varying impacts on thermal dynamics.

Based on the SHAP impact plots, NDBSI, which takes into account the effect of bare soil on LST, moderately influences the model’s predictions. Although the relationship between NDBSI and LST estimation is apparently positive in 2019 and 2024, the sign of this correlation is unclear in 2014. This fact shows a temporal effect on the relationship between NDBSI and LST, indicating that the influence of bare soil presence may vary over time. Contrasting with previous studies, this study found that the cooling effect of NDVI was not evident in 2019 and 2024. This finding is different from that found in earlier works [87,88], which emphasized the critical role of NDVI in LST modeling. This observation suggests the use of more direct variables to represent the urban greenness, such as greenspace density.

The insights gained from SHAP analysis can significantly inform urban planning strategies aimed at addressing the SUHI effect. The consistent ranking of greenspace density as a key factor in reducing LST emphasizes the need for urban planners to prioritize green infrastructure. Initiatives such as parks and green roofs can help deal with intensified urban heat stress. Preservation of urban forests is crucial for improving both air quality and thermal comfort. Furthermore, understanding the impact of built-up density on LST can guide policies towards sustainable building practices. Restriction of built-up density and increasing vegetation around buildings in identified hot spots can help mitigate the SUHI effect, enhance thermal comfort for residents, and promote overall urban sustainability.

### 6.4. Limitations of the Current Study

While this study utilized a set of 12 explanatory variables to predict LST variation, it is important to acknowledge several limitations. In addition to built-up density and greenspace density, other critical factors influencing urban landscapes, such as building height and the average patch area of green spaces, should also be considered, as highlighted by Wang et al. [24]. Precipitation [46] and population density [89] have been shown to significantly influence LST. Nevertheless, the lack of reliable data in the study area during the research periods has prevented the inclusion of these variables. Their absence might limit the ability to gain a more comprehensive understanding of the spatial variation in LST.

Moreover, this study focuses on modeling LST during the dry seasons in Da Nang’s urban center. As a result, the seasonal variations of LST have not yet been investigated. The absence of analysis for other seasons may restrict the understanding of how LST fluctuates throughout the year, potentially overlooking critical factors that influence LST during rainy seasons. In addition, another limitation of this study is that atmospheric correction was not applied to the thermal infrared data. As a result, the comparison of images acquired on different dates may be subject to uncertainties arising from variations in atmospheric conditions.

While LightGBM presents a robust data-driven approach to modeling the spatial variation of LST in the urban center of Da Nang, Vietnam, several limitations should be acknowledged. First, although LightGBM is effective, its reliance on tree-based learning may limit the model’s ability to capture certain complex spatio-temporal patterns present in the datasets. These intricate relationships might be better addressed by the incorporation of the current method with other advanced machine learning techniques, such as convolutional neural networks or long short-term memory networks. Second, as aforementioned, there are still other potential factors influencing LST that were not included in the analysis. This restriction may hinder the generalization and predictive accuracy of LightGBM.

Moreover, considering the aspect of machine learning-based modeling, the current work critically investigated the capability of LightGBM for modeling LST. While LightGBM has been demonstrated to be highly effective for the collected datasets, the capability of other advanced techniques such as Natural Gradient Boosting, Extreme Gradient Boosting, and CatBoost has not been explored.

## 7. Conclusions

Understanding the spatial distribution of LST becomes increasingly vital for effective city planning and public health initiatives. This study put forward an innovative machine learning-based method for LST within urban environments, particularly in the context of rising heatwave events. By evaluating LST variations, urban planners can better assess heat stress and develop strategies to enhance thermal comfort for residents. Utilizing LightGBM, remote sensing data, and geospatial data analysis, this study examined various factors affecting LST in the urban center of Da Nang, Vietnam, a region recently experiencing extreme heat waves attributed to rapid urbanization and climate change. The results demonstrate that LightGBM is highly effective in modeling spatial variations of LST for the years 2014, 2019, and 2024.

Notably, this study found that the model’s prediction accuracy was significantly influenced by the temporal evolution of the urban landscape. In 2019 and 2024, the data points associated with more intensive urban expansion offer greater variability in land surface temperature (LST) to the machine learning model. This increased variability enables LightGBM to better capture the urban heat dynamics, which leads to enhanced prediction accuracy. The R^2^ values are 0.92 in 2019 and 0.91 in 2024.

Furthermore, the integration of machine learning with SHAP analysis offers significant insights into how built environments influence LST variations. By identifying critical factors affecting temperature distributions, this study contributes to a deeper understanding necessary for effective urban heat mitigation strategies. This knowledge is essential for characterizing heat stress, particularly among vulnerable populations, and for implementing informed urban planning and public health measures to better cope with the adverse effects of heatwaves.

Future studies could extend these findings by applying LightGBM to model LST variations in different regions of Vietnam. Such models would support decision-making processes aimed at mitigating urban heat stress by revealing regional variations and specific factors influencing LST. It is also noted that developing predictive models that forecast future LST evolution based on projected urban development can be worth investigating. Moreover, incorporating additional explanatory variables—such as building height and average patch area of green spaces—could enhance model predictive capabilities by capturing more spatial heterogeneity affecting LST. Including demographic variables like population density and climatic factors such as precipitation would further improve the generalizability of machine learning models. Finally, exploring other advanced machine learning techniques, including gradient boosting machines and convolutional neural networks, may help identify suitable prediction models for effective modeling of LST in rapidly evolving urban landscapes.

## Figures and Tables

**Table 1:** The employed remote sensing datasets.

Dataset	Acquired Date	Bands	Resolution
Landsat 8 Level 2, Collection 2, Tier 1	3 January 2014–30 September 2014 3 January 2019–30 September 2019 3 January 2024–30 September 2024	SR_2, SR_3, SR_4, SR_5, SR_6, SR_7, and ST_B10	30 m
NASA SRTM Digital Elevation 30 m		Elevation	30 m

**Table 2:** LightGBM-based LST prediction.

Phase	Metrics	Time Period		
		2014	2019	2024
Training	RMSE	1.67	1.23	1.35
	MAE	1.30	0.94	1.02
	MAPE (%)	3.29	2.34	2.49
	R^2^	0.92	0.96	0.96
Testing	RMSE	2.30	1.64	2.02
	MAE	1.77	1.22	1.49
	MAPE (%)	4.51	3.05	3.66
	R^2^	0.85	0.92	0.91

**Table 3:** Benchmark machine learning models.

Phase	Metrics	SVM			RF			DNN		
		2014	2019	2024	2014	2019	2024	2014	2019	2024
Training	RMSE	1.89	1.53	1.92	2.13	1.49	1.93	2.32	1.47	1.83
	MAE	1.34	1.08	1.34	1.65	1.14	1.45	1.82	1.14	1.40
	MAPE (%)	3.39	2.69	3.25	4.16	2.84	3.53	4.61	2.82	3.44
	R^2^	0.89	0.93	0.92	0.87	0.94	0.92	0.84	0.94	0.93
Testing	RMSE	2.43	1.71	2.30	2.40	1.77	2.24	2.50	1.66	2.17
	MAE	1.85	1.28	1.59	1.87	1.34	1.72	1.93	1.26	1.60
	MAPE (%)	4.69	3.23	3.86	4.78	3.36	4.29	4.90	3.14	3.94
	R^2^	0.83	0.92	0.88	0.82	0.91	0.89	0.82	0.92	0.90

**Table 4:** Area of land cover classes (km^2^).

Land Cover Class	Time Period		
	2014	2019	2024
Built-up	67.97	101.11	106.51
Vegetation	102.83	100.84	98.47
Barren	55.33	26.61	22.64
Water body	13.64	11.21	12.16

**Table 5:** Fractal dimension analysis.

Year	Land Cover Category			
	Built-Up	Vegetation	Barren	Water
2014	1.6239	1.6515	1.6056	1.1529
2019	1.6874	1.6398	1.4953	1.1096
2024	1.6889	1.6351	1.4138	1.1162

## Data Availability

The code and data are available upon request.

## Abbreviations

The following abbreviations are used in this manuscript:

LST	land surface temperature
SUHI	Surface Urban Heat Island
GIS	Geographic Information Systems
SVM	support vector machine
NDVI	normalized difference vegetation index
NDWI	normalized difference water index
DNN	Deep Neural Network
RF	random forest
ANN	artificial neural network
SHAP	Shapley Additive Explanations
GEE	Google Earth Engine
TIRS	Thermal Infrared Sensor
MRF	multiplicative rescaling factor
ARF	additive rescaling factor
TPI	Topographic Position Index
NDBI	normalized difference built-up index
ANDWI	augmented normalized difference water index
NDBSI	normalized difference bare soil index
LightGBM	Light Gradient Boosting Machine
GOSS	Gradient-based One-Side Sampling
EFB	Exclusive Feature Bundling
RMSE	Root Mean Square Error
MAPE	Mean Absolute Percentage Error
MAE	Mean Absolute Error
FD	fractal dimension
